# The Quantity and Quality of Flax and Hemp Fibers Obtained Using the Osmotic, Water-, and Dew-Retting Processes

**DOI:** 10.3390/ma16237436

**Published:** 2023-11-29

**Authors:** Wanda Różańska, Barbara Romanowska, Szymon Rojewski

**Affiliations:** Institute of Natural Fibres and Medicinal Plants—National Research Institute, Wojska Polskiego 71B, 60-630 Poznan, Poland; barbara.romanowska@iwnirz.pl (B.R.); szymon.rojewski@iwnirz.pl (S.R.)

**Keywords:** flax (*Linum usitatissimum* L.) fibers, hemp (*Cannabis sativa* L.) fibers, osmotic degumming, dew retting, water retting

## Abstract

This study presents the quantity and quality of flax (*Linum usitatissimum* L.) and hemp (*Cannabis sativa* L.) fibers obtained depending on the fiber extraction method. The extraction methods used in this study were osmotic degumming, dew retting, and water retting. The degummed straw was analyzed for fiber content, while the metrological, chemical, and physical properties were determined for the fibers obtained. It was shown that these properties change based on the method of fiber extraction used. The highest fiber content in the straw was obtained using the osmotic degumming method. These fibers are characterized by a light color, no unpleasant odor, low linear mass, good tenacity, lowest hygroscopicity, and reduced flammability compared to fibers obtained via the dew and water retting of straw.

## 1. Introduction

In the textile industry, only bundles of fibers, which are the source of the raw material known as fibers, deserve attention [1]. The origin of the extraction of fibers from flax (*Linum usitatissimum* L.) dates back to the Neolithic Age, i.e., 8000 years BC. The very method of obtaining fibers from straw has been known for centuries [2]. Initially, the fibers were extracted by preliminarily retting the straw in water (otherwise known as water retting) i.e., in the Nile River in ancient Egypt, and then mechanically extracting the fibrous part from the woody parts of the plant stalk [1]. Today, in Europe, the most common process for obtaining flax or hemp fibers from straw is retting in the field, known as dew retting [3]. Despite obtaining a grey color and nonhomogeneous quality of fibers, which depends on weather conditions, the fibers are economically attractive. The use of the mechanical fiber acquisition process, i.e., mechanical decortication, is also becoming increasingly popular [4,5]. However, a more advantageous separation of non-cellulosic components from the fibers is achieved via the pre-treatment of the raw straw material. In the process of obtaining fibers from bast plants, such as flax (*Linum usitatissimum* L.) and hemp (*Cannabis sativa* L.), one should distinguish methods such as biological (water retting, dew retting, and enzymatic treatment), chemical, and physical, as shown in Table 1.

### 1.1. Mechanical Processing

In mechanical processing, the method of separating fibers from plant tissues depends on the further use of the fibers [1,5,6,7,8,9,10,11,12,13], i.e., for textile or non-textile purposes.

The following mechanical processes are used for textile purposes:

- Scutching—a braking and scutching turbine known as the Deporter turbine, which is still in use today;
- Hackling to remove short fibers and other plant tissues, such as the shives;
- Cottonization is the process of further dividing the fibers into elementary fibers, resulting in a thinning of the fibers. The resulting fibers are similar in length and spinning properties to cotton fibers.

For non-textile purposes, decortication is used. Decortication is the mechanical removal of non-fibrous matter from raw straw, also known as green decortication. According to Schilling and Muller [1], the decortication of straw contributes to fibers weakening. As the dry straw passes through the crushing rollers, a number of transverse fiber cracks are created, reducing the strength of the fibers. The disadvantage of obtaining fibers using mechanical methods alone is the poor divisibility of the fibers. Hackling produces a large amount of waste and the resulting yarn is hard and has reduced strength. In addition, the efficiency of the process is greatly influenced by the moisture content of the straw [2]. The optimum moisture content should be 14–16%, but below and above these values the efficiency of the decortication process decreases.

Harry Gilbertsen developed a laboratory decorticator for processing both retted flax straw (for seed purposes), nettle, and unretted hemp straw. David Bruce’s team then developed a prototype of an industrial-scale decorticator [4].

The Institute of Natural Fibres and Medicinal Plants National Research Institute (INF&MP-NRI) in Poznan also started work on the extraction of lower-quality fibers with the possibility of using them in the pulp and paper, composite materials, energy, and construction industries [5]. For this purpose, INFMP-NRI has developed a technological line that allows obtaining fibers from straw using the decortication method without prior drying of the straw [5].

### 1.2. Biological Methods

Biological methods of fiber production include the following:

- Retting the straw in the field, common name dew retting;
- Water retting.

The traditional method of separating the fibers from the woody parts of the plant stalk and obtaining and cleaning it from non-cellulosic components is the retting of fibrous plants on the surface of an agricultural field, known as dew retting [1,5,6,8,9]. This method is still used today, mainly for economic and environmental reasons. The essence of the process is that micro-organisms, mainly fungi (*Cladosporium herbarum*, *Mucor* sp., *Rhizopus* sp. and *Epicoccum nigrum*), develop on the spread stems and their growing mycelia with their hyphae penetrate the stem and secrete enzymes (ferment) that break down pectin substances, commonly known as plant glue. This process is difficult to control and, depending on weather conditions (season, air temperature and humidity, amount of rainfall, etc.), fibers of different quality are obtained [14,15]. During retting, flax straw changes color from yellow-green to steel-grey. The fibers obtained are grey in color and do not have the unpleasant odor of volatile fatty acids produced during water retting. Bacterial growth causes the straw to turn light and creamy, while exposure to sunlight and lack of moisture causes it to rust.

In Europe, the retting process of flax in the field takes 28 to 41 days, depending on the region and weather conditions. The optimum parameters are a temperature of 15 to 20 °C and a humidity of 60%. The shortest retting time was observed when the average temperature was 15 °C and rainfall was about 60 mm. The longest retting time was 70 days at an average temperature of 11.5 °C and rainfall of about 32.6 mm.

Flax should be harvested at the green-yellow stage before the seeds are fully ripe, during a period of low rainfall. This helps to reduce the rotting of the straw during retting. The moisture content of retted straw should be less than 15%, but if it is higher, losses may occur during storage [15].

In order to speed up the dew-retting process, the following methods can be used: nitrogen fertilizers, breaking straw in the root part, and the use of desiccants (Purivel, Roundup). The use of nitrogenous media to accelerate the development of microflora on the surface of the stems, and thus the separation of fibers from woody parts of the plant stalk, has also been the subject of research [1]. Desiccation involves the use of herbicides prior to straw harvesting in order to accelerate the ripening of the plant, which improves the dew-retting process. The use of pesticides or herbicides can cause selective development of the microflora and sometimes create unfavorable conditions for the dew-retting process.

Improving the quality of the retting process in the field can also be achieved by using fungal solutions [16]. The best results were obtained when the fungal mixture was applied the first time immediately after straw removal and the second time immediately after turning the straw. The best results were obtained when the fungal mixture was applied the first time immediately after harvesting the straw and the second time immediately after turning the straw. Field retting is still used on an industrial scale by fibrous plant fiber producers.

The water-retting process is carried out using various methods. Among other things, retting is carried out in natural water reservoirs and in retting ponds [1,5,6,8,9,10]. The simplest and most primitive method was retting in pits with cold water. Another method was retting in ponds and rivers, such as the River Lys in Belgium [1]. The retting method was also used on an industrial scale in Germany and Finland.

Retting in warm water was first described by Schenk in Ireland in 1846 and then spread rapidly throughout Europe. Retting was carried out at a temperature of 30–32 °C using condensation water from a steam machine. The resulting fibers were light, hard, and not very durable. It was not until canal retting according to Dr H. Schneider’s system that flax could be retted on an industrial scale [1].

The most modern and best system of retting is retting in basins [1,5,6,7,8,9,10,14,15]. The process of cold and warm water retting is based on biochemical phenomena occurring under the influence of bacteria such as *Bacillus amylobacter*, *Bacillus felsineus*, *Granulobacter pectinovorum*, *Clostridium felsineum* and *Bacillus comesii rossi*, which cause the fermentation of pectin substances and the separation of woody parts of the plant stalk from the fibers. In the classical straw retting method, the microorganisms that cause pectin fermentation enter the retting liquid along with the stalks, soil particles, liquid, air, and reused liquid. This process can take place under aerobic or anaerobic conditions. Warm water retting, carried out under controlled conditions (temperature and pH), makes it possible to shorten the process compared with cold water retting. The process of retting flax in warm water takes 70–100 h. The fibers obtained are characterized by light color, soft handle, and a characteristic, unpleasant odor due to volatile fatty acids formed during water retting [14,15].

To accelerate the pectin decomposition process, degumming stimulants were used in the form of nitrogenous media (potassium, magnesium, and phosphorus salts and urea) or leavening agents [17]. In addition, fluid recirculation, double retting, and the addition of retted wrack bedding or enzymes were used [18,19]. The aim of these activities was to achieve faster and simultaneous degumming of the fibers that cause the fermentation of pectin substances and the separation of woody parts of the plant stalk from the fibers and the decomposition of the pectin in the fibers itself, which contributed to shortening the retting process and improving its quality, mainly by increasing the divisibility and thinning of the fibers bundles. Anaerobic retting with the use of anaerobic bacteria, *Clostridium corallinum,* and aerobic retting [20,21,22,23,24] with the participation of bacterial leaven [25] and the addition of urea [26] and nitrogen media [23] have also been used to accelerate the process [24]. The classic warm water-retting technology for flax and hemp straw, which has been used in the retting industry, required large amounts of water for proper operation, resulting in highly polluted retting effluent at various stages of the process [10].

In total, the composting effluent consisted of post-composting effluent, effluent from the rinsing of straw that had grown back in ponds, and post-wringing effluent. The post-retting effluent was reddish-brown in color, turbid, with an unpleasant odor of decomposing pollutants, mainly organic compounds, the products of which were mainly volatile fatty acids formed during fermentation (such as acetic acid, butyric acid, propionic acid, formic acid, and valeric acid). In addition, the effluent contained protein degradation products, mainly amino acids. The effluent after fermentation was characterized by significant biological oxygen demand (BOD) values, at the level of 870–3500 O_2_/dm^3^ for flax and 1330–3860 O_2_/dm^3^ for hemp [27].

Today, warm water flax retting is used in Egypt, where natural climatic conditions are used to heat the water in the tank. Sheaves of flax straw are placed at the bottom of the tank and weighted down with stones to prevent them from floating out. This process has been abandoned in most European countries (except Bulgaria and Romania) for economic reasons and because of the nuisance of the effluent.

### 1.3. Chemical Degumming

In chemical degumming, the fibers are separated by the action of chemicals. No microorganisms are involved.

Chemical compounds such as acetylene, acetic acid, sodium chlorite, oxidized water, sodium carbonate, and others are used in the chemical extraction of natural fibers [1,9,28]. The purpose of chemical degumming is to reduce the processing time (even to a few hours), but it can reduce the strength and color of the fibers obtained. The process must be monitored to prevent damage to the fibers. Although chemical degumming methods for fiber plants have been known for a long time, they are rarely used today. The main factors limiting the use of chemicals are their high cost, as well as their environmental impact, and the need to purchase suitable equipment. The requirements for the chemical process are as follows:

- To not disturb the structure and properties of the fibers—cellulose;
- To preserve the waxes and fats responsible for the softness of the fibers and the pectin that binds the elementary fibers into bundles;
- Destruction of the tissue connecting the phloem to the woody parts of the plant stalk;
- Easy removal of the woody parts of the plant stalk from the phloem.

The use of 5% oxalic or sulphuric acid provided a long fiber yield of 17%. The fibers obtained were rough to the touch and lacked luster. In addition, the quality of the fibers was strongly influenced by the concentration of the acid solution used. A lower concentration of acid made it more difficult to separate the fibers from the woody parts of the plant stalk, while a higher concentration of acid weakened the fibers [2].

The disadvantage of degumming straw with alkali (sodium hydroxide or caustic soda) includes swelling of the adhesive substances, making them difficult to rinse out, with the fibers stuck to the woody parts of the plant stalk again after drying [1].

In degumming with acids and alkalis, the method involved treating the raw material successively with acids and alkalis, neutralizing the process after each application. The result was fibers with improved quality, luster, and softness. Compared to fibers obtained using the retting method, the chemically degummed fibers showed a deterioration in strength, which resulted in higher losses during further processing [1].

In the case of removal using oxidizing agents (hydrogen peroxide), the removal method proved to be too radical and unprofitable. Oxidizing agents affected all straw components [1].

Although chemical methods have been known for years, they are not currently widely used. The use of chemicals in the degumming process is difficult due to their high cost, environmental impact, and the need to purchase specialized equipment.

### 1.4. Physical Methods

It is also known that bast fibers can be degummed by the action of physical agents on the straw, such as ultrasound, steam treatment, electromagnetic radiation, electro-osmosis, and osmosis.

On an industrial scale, in earlier years, steam treatment of hemp straw was used [10,29]. The essence of the straw steam treatment consists of the hydrolysis of substances that glue together bundles of fibers in the stem and steam under pressure in an autoclave. Hydrolysis is favored by high moisture of the stems, so before evaporation, the straw is soaked in warm water (for about 30 min) and then evaporated in an autoclave (at a pressure of 0.2 bar for about 2.5 h). As a result of hydrolysis, the connections between the bast fibers bundles and the woody parts of the plant stark, and the fibers can be easily separated.

In 1998, R.W. Kessler [30] used the steam explosion method for degumming flax fibers as an alternative process to the dew-retting method. According to the author, steam treatment of flax fibers makes it possible to obtain good-quality fibers with small quantitative losses. The minimum period of steam explosion was 2 min. Prior to steam treatment with sodium hydroxide, pre-treatment allows to compensate for poor straw degumming and therefore to obtain more good fibers. Steam treatment produces short fibers for textile and composite applications.

Garcia-Jaldon et al. [31] used steam treatment in degumming off the decorticated hemp fibers. The authors showed that pectin and hemicellulose are hydrolyzed and become soluble in water and bases, making the fibers easier to degum. The best conditions were the use of alkaline and steaming treatment under the following conditions: time of 90 s, temperature of 200 °C, and pressure of 1.5 MPa. The steam treatment process is fast and controllable, cheap, and suitable for the use of hemp fibers. In addition, it is a good alternative to the process of retting the straw on the field.

Another method of physical retting developed by Migoni and Allessandro Bozzini [32] is using electric resonance electrodes in a pool of water to speed up the retting process. The use of special electrodes allows for the formation of an electric field without current flow, which causes the creation of a resonance that breaks pectin bonds, leading to the degumming of the fibers. The method can be supplemented with the addition of pectinolytic enzymes, which further reduce the retting time. The fibers obtained are of good quality, with a light color, and the process is not accompanied by the development of bacteria; thus, the unpleasant smell of degummed fibers is absent.

In addition to the enzymatic processing of flax fibers, the degumming process was also supported using ultrasound. The research was carried out in the experimental plant on a larger scale using the laccase enzyme with a 1% concentration in an ultrasonic bath under the following processing conditions: at a time of 25 min and ultrasound frequency of 35 kHz [33]. The authors showed that such a combination of processes allows obtaining qualitative fibers at the level of the cotonized fibers from a physico-mechanical point of view. Also, a study conducted in 2015 [34] showed a positive effect of the combination of enzymatic degumming and ultrasound on the quality of flax fibers.

An interesting solution for obtaining fibers is the use of microwaves in the retting process. For this purpose, flax [35] and hemp [36] straws were retted in water (at different times) and then microwaved, determining the effect of the applied power and exposure time on the quality of the fibers. The authors showed that retting helps loosen the bonds of pectin bonds connecting the fibers to the woody parts of the plant stalk in the plant. However, the use of microwaves caused pressure in the stem, which affected its disruption and separation of fibers from the rest of the plant. Optimal retting for both flax and hemp was obtained using retting at 24 h and exposure to 2 W/g microwaves at 20 min.

Another solution for obtaining the fibers was to use a radio frequency (RF) heating system [37]. The authors used a radiofrequency generator to improve flax retting in water. Retting was carried out in distilled water during 48, 144, and 240 h. Then, flax straw samples were placed between two electrodes in a generator and heated by creating a radiofrequency electric field. Three variants were used in the degumming process, with varying temperatures of the degumming process and the power of the waves used, i.e., 40 °C and 200 W, 65 °C and 350 W, and 90 °C and 450 W. The authors showed that the use of an RF generator for 60 min at 40 and 90 °C temperatures resulted in the maximum improvement in the retting efficiency of fibers compared to water-retted fibers, which slightly changed the color of fibers.

INF&MP-NRI focused on a new method of degumming fibers to obtain higher-quality raw material. The method of fiber extraction was based on the use of physical phenomena, diffusion, and osmosis [38,39], occurring in contact with water inside fibrous plants. It gives the possibility of extracting fibers from plants without influencing the natural characteristics of the degummed fibers. As a result, the fibers obtained were devoid of the characteristic odor, as well as non-degraded (durable) and perfectly degummed, free of impurities [40,41]. Currently, this method has been implemented to evaluate progress in the cultivation of fibrous plants, and in the determination of fiber content in fibrous plants.

**Figure 1:**
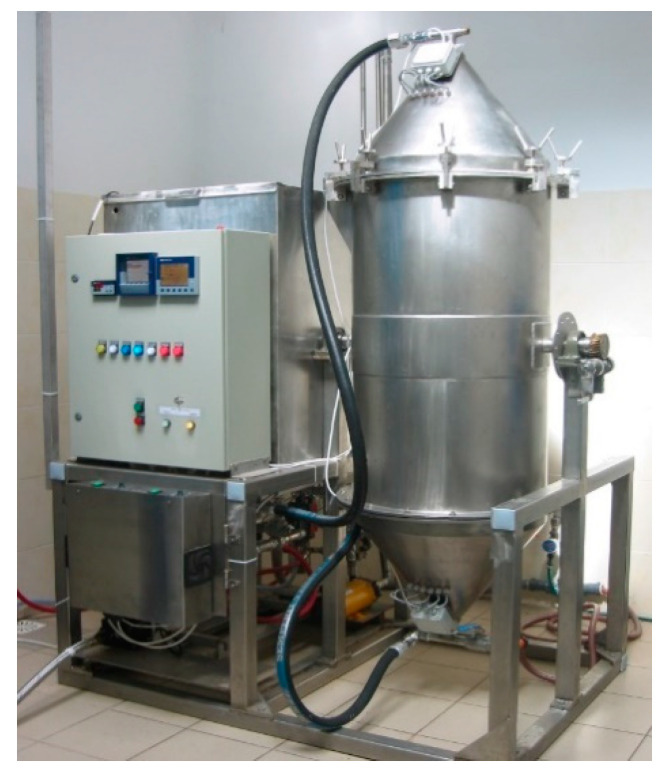
Prototype device for osmotic degumming of fibers in a periodic raw material loading system, built using LIST in Poland.

**Figure 2:**
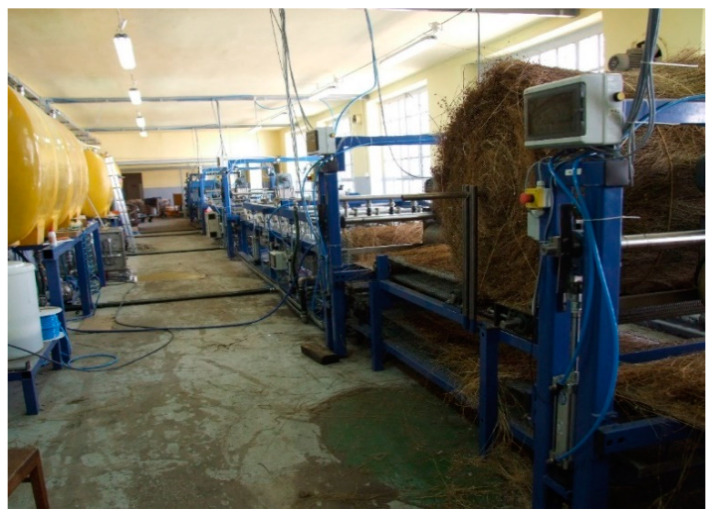
Prototype device for osmotic degumming of fibers in a continuous raw material loading system, built using LIST in Poland.

In cooperation with the Łukasiewicz, Institute for Sustainable Technologies (LIST), in Radom, a large-scale device for degumming fibrous plants in the system of periodic operation was designed and built (Figure 1) [42]. The device allowed to processing of raw material in the amount of 20 kg of hemp and 15 kg of flax straw. The raw material is loaded through an openable collector). In the next stage, a prototype of the device for the osmotic degumming of bast plant fibers in the system of continuous operation was created (Figure 2) [43].

The module consisted of a bathtub equipped with a straw transport unit, an ultrasonic inductor, and a shower, harrow, and feeding unit. Sequentially connected modules form a processing line. The water flows through the modules in countercurrent to the direction of the straw. The amount of water in a module in a closed circuit is 3 m^3^. The power supply device was adapted to straw baled in a roll during straw harvesting in the field, Figure 2 [43].

The study also investigated the effect of lengthwise and crosswise water flow through the fibers straw at different process parameters [44]. The research has shown that the process involving the lengthwise flow of liquid through the raw material results in better fiber quality, i.e., fibers with lower linear weight and higher strength compared to crosswise flow like the classic water-retting method.

In addition, a characterization of the physical phenomena that occur during the physical–mechanical process of degumming the fibers from flax straw was presented in [45]. For this purpose, an analysis of the mass movement was carried out, using the rules of fluid mechanics that occur during fluid flow through the raw material. It was shown that the use of forced water flow accelerates the exchange of mass in the process. Also, the increase in temperature intensified the process, which is associated with lower water consumption and an improvement in the quality of the fibers obtained.

This research is a continuation of previous work on obtaining fibers using the osmotic degumming method, which presents in detail the mechanism of the osmotic degumming process in comparison with the field and water-retting method [45].

This work focused on assessing the quantity and quality of fibers obtained using three extraction methods, i.e., dew retting, water retting, and osmotic degumming. The osmotic degumming method uses an apparatus with a periodic raw material loading system, while the water-retting method uses a bathtub imitating a retting pool. The same process conditions were maintained for both methods, i.e., temperature 30 °C and time 72 h for flax and 144 h for hemp. However, in the dew-retting method, straw was spread on the experimental plot in parallel, and the retting process was controlled until complete retting. The selection of conditions for the osmotic degumming and water-retting method was based on the optimal conditions available in the literature [44,45].

The research conducted will be used to apply the obtained fibers in composites and to evaluate the obtained composites, which will be presented in a future article.

## 2. Materials and Methods

### 2.1. Materials 

For this study, we used Flax (*Linum usitatissimum* L.) and Hemp (*Cannabis sativa* L.) straw. Flax straw of the Dutch Agatha (NL) variety was collected from Baird Poland sp. Zoo in Bolkow, Poland. Hemp straw of the Bialobrzeskie (PL) variety was collected from the Lenkon Experimental Station in Steszew, Poland.

The straw obtained was from industrial plantations grown for fibers. In the case of flax grown for fibers, 120 kg/ha of seed was used, while in the case of hemp, 60 kg/ha of seed was used. Flax was harvested at an early stage of yellow maturity, while hemp was harvested at an early stage of hemp seed maturity.

To obtain fibers from straw, whole flax straw was used. In the case of hemp stalks, due to their average technical length of 184 cm), they were cut in half at 87 cm ± 5 cm. The cutting of the stalks was dictated by placing them in devices for degumming, retting, and mechanical processing, which allowed for the processing of straw no longer than 100 cm.

### 2.2. Degumming Methods

#### 2.2.1. Osmotic Degumming

The studies on fibers degumming by osmosis were carried out on a large laboratory scale using the device in a periodic raw material loading system.

**Figure 3:**
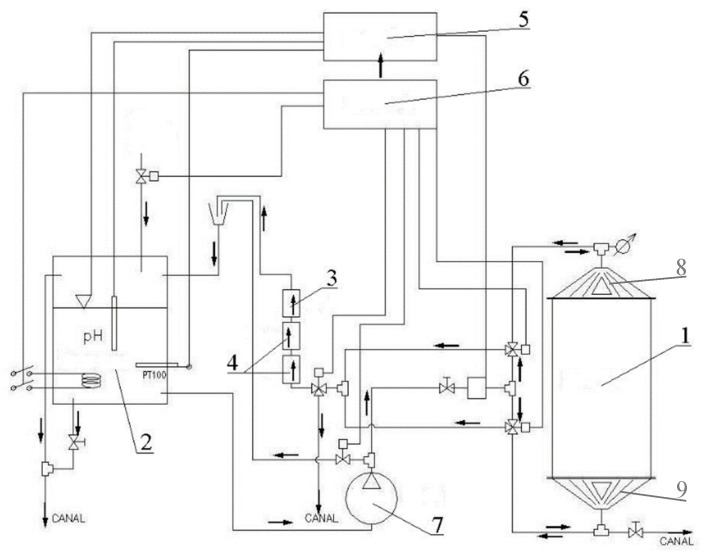
The schema of an osmotic degumming device in a periodic raw material loading system, built by LIST in Poland [42].

The device was designed and built in cooperation with the LIST in Radom (Figure 1). The installation included the following (Figure 3):

1. Reactor—working tank, the upper and lower collectors of which were equipped with a 30 kHz ultrasonic generator;
2. Reservoir—a reservoir for soft process water, equipped with a heater with temperature sensor, pH electrode, and water level sensor;
3. A lamp type C UV source to prevent the development of retting bacteria during the process;
4. A filtration unit for the closed cycle of the process liquid;
5. A controller;
6. An installation for steering and diagnostics for setting the parameters and online recording of the physical parameters;
7. A pump for forcing the flow of the water and other hydraulic and control installations;
8. An openable collector equipped with ultrasound;
9. A permanent collector equipped with ultrasound.

The straw was placed in the reactor through an openable collector. Only flax straw, which is much wider in the root part, was put into the reactor alternately, first with the root part and then with the top part. In this way, it was possible to load the reactor with 15 kg of flax straw. The straw was then flooded with tap water, which was pumped into the reactor from the reservoir. In order to obtain comparable penetration conditions of the water flowing through the entire volume of the batch in the reactor, the reactor was equipped with appropriate collectors (upper and lower) that distribute the water evenly over the entire cross-section of the reactor.

Our earlier work [45] showed that when the raw material is loaded with 15 kg of flax straw, the hydromodule of straw to water in the reactor is as much as 1:10 (1 kg of straw per 10 dm^3^ of water). During the osmotic degumming process, the water in the tank was heated to a preset temperature. In addition, in order to standardize the conditions of interaction of water with straw and degummed fibers, a periodically (every 2 h) variable direction of flow of technological liquid through the straw inserted into the reactor was used, i.e., once through the opening collector, and then through the permanent collector. The straw was subjected to the degumming processes under controlled conditions (Table 2).

The degummed straw was subjected to a hydrodynamic rinse using cold water. Then, excess water was removed on a laboratory wringer. The degummed straw was dried in a laboratory dryer at 60 °C (±5 °C) for 48 h.

#### 2.2.2. Dew Retting

After harvesting, flax straw was spread evenly in the field and turned over periodically to ensure even straw retting. The evaluation of the degree of retting was performed organoleptically by breaking and removing the woody parts of the plant stalk (common name: shives) by hand from the stem. If the straw could be easily broken and the fibers were separated from the claws after breaking, the retting process was complete. Next, the straw was collected from the field and dried.

#### 2.2.3. Water Retting

**Figure 4:**
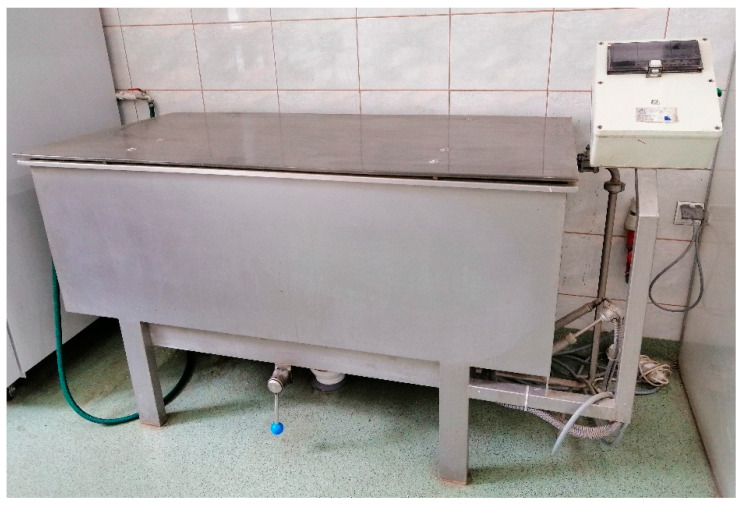
Prototype device for water retting of straw, built by LIST in Poland.

The straw was placed in a tank (Figure 4) parallel to each other, secured against flowing out, and flooded with water. The retting process was carried out at a water temperature of 30 °C, a ratio of 1:10 (1 kg of straw per 10 dm^3^ of water), process time of 72 h for flax, and 144 h for hemp. Laboratory tests included 3 kg of raw material. Next, the straw was rinsed using cold water and dried in a laboratory dryer at 60 °C air temperature for 48–72 h. The mechanical processing of the degummed straw was carried out using the laboratory scutching unit.

### 2.3. Mechanical Processing of Degummed Straw

The mechanical processing of the degummed straw was carried out on the following:

- Laboratory breaking machine—due to the thickness of the stalks, only the hemp straw was broken, built by Czech Flax Machinery (CFM) in the Czech Republic;
- Laboratory turbine, built by Czech Flax Machinery (CFM) in the Czech Republic.

### 2.4. Test Methods

#### 2.4.1. Straw Evaluation

Flax and hemp straws were tested according to PN-P-80103:1996 [46] (fiber flax straw) by assessing the length, thickness, color, attitude, and healthiness of stems.

#### 2.4.2. Fibers Evaluation

Flax and Hemp fibers were tested according to appropriate standards to evaluate structural and mechanical parameters:

- The mass of extracted fibers (%) was tested using a gravimetric method. Fibers were mechanically extracted and weighed. The result is presented as the mass of fibers contained in 100 g dry weight of the raw straw sample (g/100 g).
- Linear mass of the fiber (tex) test was performed with the use of the gravimetric method according to the Polish Standard PN-EN ISO 1973:2022-03 [47]. A 10 mm fibers section was cut off from the middle parts of the flax fibers and then formed into bundles containing 100 pieces of fibers. The measurement of the mass of separate bundles allowed for the determination of the mean linear mass of fibers. The test was carried out under ambient conditions.
- Tenacity (cN/tex) was determined according to the Polish Standard PN-P-04676:1986 [48]. The tenacity was tested by breaking the fiber bundles of specific linear mass with an Automatic Tensile Tester—STATIMAT ME from TXTECHNO, DE (the distance between clamps was 3 mm).
- Microscopic analyses—the micrograph images of longitudinal views and cross sections of flax fibers were made with a scanning electron microscope (SEM) using Hitachi S-3400N, manufactured by Hitachi, Ltd., Tokyo, Japan, in a high vacuum mode (a secondary electron detector SE). Fibers were sputtered with gold. A magnification of ×250, setting the table height to 20 mm, and an accelerating voltage of 20 kV were used to observe the characteristic features of the fibers’ surface.
- Wax and fat content (%) was measured according to the Polish Standard no. BN-86/7501-10 [49]. The percentage content of wax and fat substances was determined by extracting them using an organic solvent (petroleum ether) in a Soxhlet extractor and weighing the residues after the vaporization of the solvent.
- Lignin content (%) was determined according to the Polish Standard BN-86/7501-11 [50]. The lignin content was measured by dissolving cellulose, hemicellulose, and pectins with a mixture of concentrated sulphuric and orthophosphoric acids, followed by draining off the remaining insoluble lignin.
- Pectin content (%) tests were conducted using a gravimetric method developed at INF&MP-NRI. The percent share of pectins was determined by dissolving them in ammonium citrate and then precipitating them from the solution with calcium chloride and by measuring the weight of the calcium pectinate precipitated from the solution.
- Cellulose content (%) in flax and hemp fibers was measured according to the Polish Standard no. PN-P-50092:1992 [51]. The cellulose content was measured by dissolving lignins and other substances present in the fibers with a mixture of acetylacetone and dioxane, acidified with hydrochloric acid.
- Hemicellulose content (%) in the flax end hemp fibers was determined according to the Polish Standard BN-77/7529-02 [52]. The hemicellulose content was measured by dissolving the hemicellulose present in the fibers using a 1% solution of sodium hydroxide, filtering off the residue after dissolution, drying it, and weighing it. Then, the hemicelluloses were calculated from the mass loss of the sample.
- Fiber hygroscopicity (%) was measured under conditions of 65% and 100% relative air humidity, according to the PN-P-04635:1980 standard [53]. The hygroscopicity was determined as a quotient of the mass increase that the bast fibers undergo when placed for a specified time in the air with 65% and 100% relative humidity, respectively, and the mass of the fibers after drying.
- Combined analysis TGA-FTIR—TGA. Thermogravimetric analysis (TGA) was performed using a TA Instruments Analyzer Q50 (TA Instruments, New Castle, DE, USA). A test sample (approximately 15 mg) was heated in the temperature range of 30 to 700 °C at a heating rate of 10 °C/min in a nitrogen atmosphere at a constant gas flow rate of 90 mL/min.
- During the TGA study, the gases released were identified using Fourier Transform Infrared Spectroscopy (FTIR). The tests were performed on a TA Instruments iZ10 model, Thermo Fisher Scientific, Madison, WI, USA. The spectrum of the released gases contained 8 scans per second at a resolution of 4 cm^−1^ in the range of 600 to 4000 cm^−1^.
- ATR-FTIR Analysis—Fourier transform infrared spectroscopy (FTIR) with an Attenuated Total Reflectance (ATR) attachment was performed with an iS10 model instrument (TA Instruments, New Castle, DE, USA). The spectrum of the fibers contained 8 scans per second at a resolution of 4 cm^−1^ within the range from 600 to 4000 cm^−1^.
- Flammability tests were carried out via a pyrolysis combustion flow calorimeter (PCFC) from FTT, UK. Tests were performed according to the standard of ASTM D7309-2007 [54]. The heating rate was 1 °C/s. The pyrolysis temperature range was 75–750 °C, and the combustion temperature was 900 °C. The flow was a mixture of O_2_/N_2_ 20/80 cm^3^/min, and the sample weight was 3–4 mg. The maximum heat release temperature (Tmax) and maximum heat release rate (HRRmax) were determined.
- Statistical analysis was calculated based on significant differences between fibers that were assessed using one-way analysis of variance (ANOVA) and Tukey’s Honest Significant Difference (HSD) test, and *p* < 0.05 was considered a significant difference.

## 3. Results

**Figure 5:**
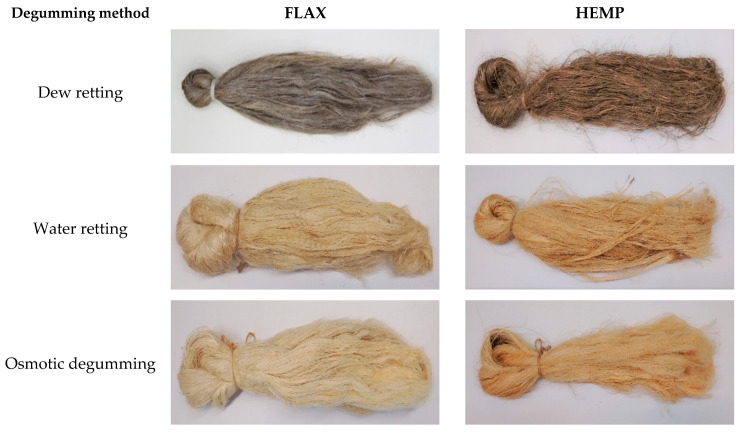
Long flax and hemp fibers.

The tests showed that using flax and hemp straw (Table 3) in the dew-retting method produced grey-colored fibers (Figure 5).

However, the water-retting and osmotic degumming methods yielded light-colored fibers. As the authors of [10,55,56] report, the color transition depends on the extraction method, where the straw becomes lighter under the influence of bacteria, involved in water retting, and darker to steel gray or brown under the influence of fungi, involved in dew retting. Therefore, the light color of the fibers obtained in the osmosis process can be explained by the fact that during the osmotic degumming process, there is no influence of microorganisms on the straw; therefore, the natural color of the obtained fibers does not change. In addition, the osmotic degummed method yielded fibers characterized by the absence of an unpleasant odor compared to the water-retting method, which is due to the presence of volatile fatty acids [1,8,9,10].

**Figure 6:**
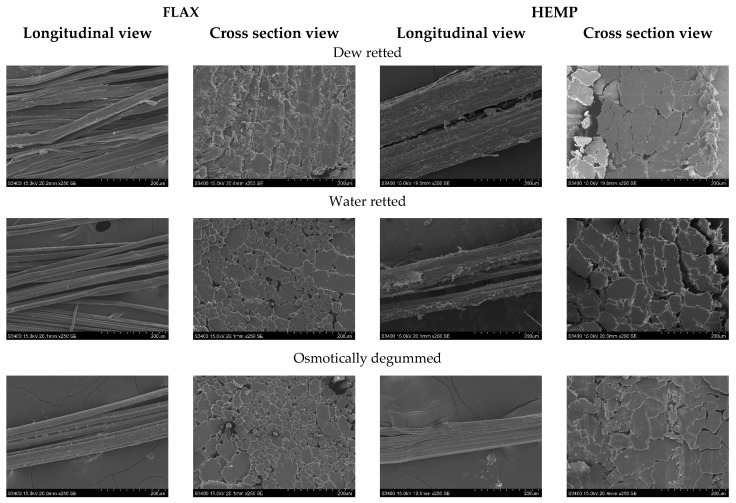
View of flax and hemp fibers. Scanning Electron Microscope (SEM), 250 magnification.

Investigations into the morphological structure of the fibers clearly showed changes in the degree of fiber elementarization in the technical fiber bundles (Figure 6). In all cases, changes in the shape and size of the cross-section of the elementary fibers were observed. The microscopic images of the cross-section (Figure 6) show that hemp fibers, as well as flax fibers obtained using the straw method, have the lowest fiber bundle divisibility compared to other fibers. However, the analysis of the photos with a longitudinal view showed that flax fibers obtained via osmosis and soaking are characterized by a lower amount of visible impurities compared to linen fibers. In the case of hemp, only the fibers obtained via osmosis are characterized by fewer visible impurities compared to the other fibers. In addition, the surface of all fibers tested was shown to be smooth and undamaged.

The highest mass of extracted fibers in the total mass of straw was obtained using the osmotic degumming method for both fibers, i.e., 38.12% for flax fibers (including 29.64% long fibers and 8.48% short fibers) and 35.56% for hemp fibers (including 26.40% long fibers and 9.16% short fibers) compared with the fibers obtained using other extraction methods, which was statistically significant only for hemp fibers (Table 4). Only hemp fibers obtained using the retting method had the lowest fiber content in the straw. This can be explained by the more destructive effect of the dew-retting method compared to the water-retting method, which resulted in a lower fiber content during mechanical processing. According to the literature, the dew-retting process is difficult to control and depends on the weather conditions (season, air temperature and humidity, amount of rainfall, etc.), and the fibers obtained are not homogeneous [14,15].

The tenacity of the fibers obtained using the osmotic degumming method was at a similar level to that of fibers obtained using the water-retting method, with a minimum difference of 0.01 cN/tex for hemp, whereas for flax it was 3.31 cN/tex lower (Table 5).

The study showed that the tenacity value of both flax (13.88 cN/tex) and hemp (4.59 cN/tex) fibers obtained using the dew-retting method was lower than that of other fibers, which was statistically significant (Table 5). As mentioned above, this can be explained by the fact that the dew-retting process is more destructive to the extracted fibers.

The analysis of the results for the linear mass of the hemp fibers showed that the osmotic degumming of straw can obtain more divided fiber bundles at the level of 0.8 tex than the fibers obtained via the water retting of the straw with a value of 1.1 tex, which is statistically significant. In the case of flax fibers, no differences in linear fiber mass were found for all extraction methods tested.

An analysis of the chemical composition of the fibers tested made it possible to determine the content of the main component, i.e., cellulose, and the accompanying components, i.e., waxes and fats, lignin, pectin or hemicellulose, depending on the method used to extract the fibers from the straw, as shown in Table 6.

According to the literature [1], the cellulose content of flax fibers varies between 64 and 84% and that of hemp fibers between 67 and 78%, whereas the lignin content varies between 0.6 and5% for flax and 3.5 and 5.5% for hemp pectin, and the hemicellulose content varies between 19% for flax and 17% for hemp.

Pectin plays an important role in flax fibers, acting as a glue to bind the fibers together and giving them luster and softness. Two fractions of pectin can be separated in fibrous plants: the water-soluble fraction A and the water-insoluble fraction B [1,5,8,9]. The correct removal of pectin substances in the pre-treatment process determines the separability and thus the linear weight of the fibers and the suitability for spinning [2].

During the retting process, pectin A, which is broken down by bacteria and fungi, is most easily removed. Pectin B remains in the fibers and, together with other compounds, determines its cohesiveness. The excessive removal of pectin makes the fibers unpleasant to the touch, dry, and rough. Its complete removal, on the other hand, causes the fiber bundles to break down into elementary fibers [1].

From a technological point of view, waxes and fats are also important ingredients. They give the fibers a soft handle, a low coefficient of friction, and therefore an ease of movement. In flax straw, waxes are mainly found on the outside of the stalk, in the cuticle, and to a lesser extent in the fiber cells [1,5,8,9].

In the plant, lignin plays a crusting role in the amorphous areas of the cellulose, giving it stiffness. In the elementary fibers, lignin is found in the primary wall and the outer part of the secondary wall. It is an undesirable substance in processing. It reduces the grip and flexibility of the fibers. It makes the fibers more brittle and fragile, thereby decreasing the strength and elasticity parameters. In addition, the lignin content reduces the divisibility of the fibers [1,5,8,9]. 

The detailed analysis of the chemical composition results of flax fibers obtained via water retting and osmotic degumming have been shown to have a lower lignin, pectin, and hemicellulose content than those obtained using the dew-retting method, which was statistically confirmed, as shown in Table 6. This indicates that fibers degumming in water such as water retting and osmotic degumming results in better removal of non-cellulosic components from the fibers compared to the dew-retting method. In the case of hemp, the statistically lowest lignin and pectin content was obtained for the water-retted fibers compared to other fibers. The statistically lowest value of hemicellulose was obtained for osmotically degummed hemp fiber compared to other fibers.

The analysis of the results for both flax and hemp fibers showed that the hygroscopicity at 65% moisture content (Table 7) of the fibers depends on the linear mass of the fibers (Table 5). It can be concluded that as the linear mass of the fibers increases, the hygroscopicity of the fibers increases. However, this relationship was not statistically significant.

Analysis of the flax fibers’ hygroscopicity results for both 65% and 100% moisture content showed that the fibers obtained using the dew-retting method were characterized with a higher hygroscopicity, compared to the fibers obtained using water retting with the lowest hygroscopicity, which was statistically significant (Table 7). The hygroscopicity at 65% moisture content of the dew-retted fibers was more than 0.50–0.64% higher than that of the other fibers, which differed by only 0.14% between them. For hemp fibers, for 100% moisture content, a similar relationship to that of flax was shown, while for 65% moisture content, the highest hygroscopicity was that of the fibers after the water-retting method and the lowest was that of the osmotically degummed fibers. The hygroscopicity at 65% moisture content of the fibers soaked in water was higher by more than 0.23–1.04% compared to the other fibers, which differed by 0.81% between them. According to Cousins [57,58], mechanical properties decrease with the absorption of moisture in the fibers. Our research has shown that this relationship can only be observed for flax fibers; in the case of hemp, the fibers obtained using the water-retting method were characterized by both the highest specific tenacity and hygroscopicity, compared to the other methods of obtaining the fibers.

**Figure 7:**
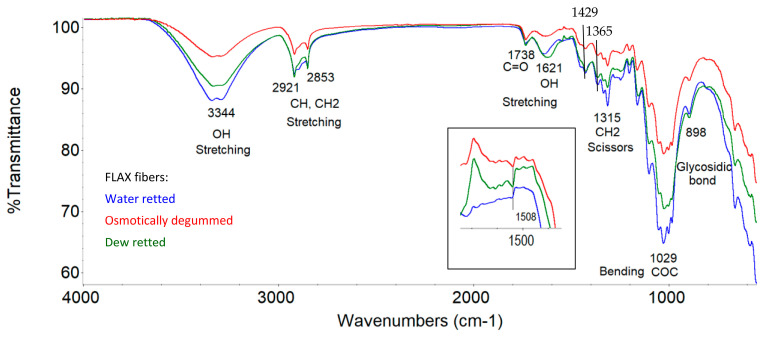
ATR-FTIR spectra of flax fibers.

**Figure 8:**
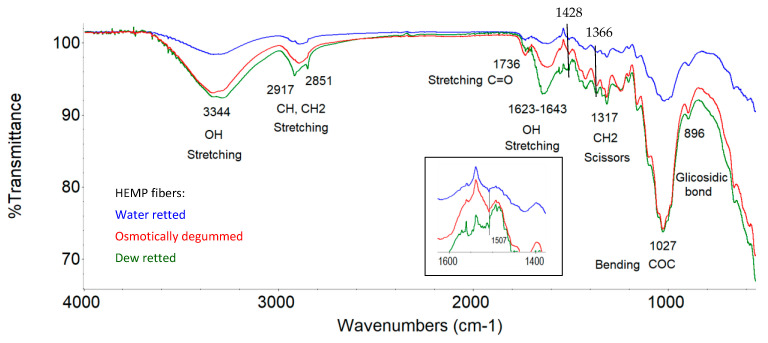
ATR-FTIR spectra of hemp fibers.

Based on the infrared spectrum obtained (Figure 7 and Figure 8), the occurrence of bands in the wavelength range that correspond to the bond vibrations of functional groups, such as OH, C=O, C=C, COO, CH, CH_2_, CH_3_, COC, and C-O, was identified in all the fibers studied.

An analysis of the fiber structure showed that all identified bands come from the chemical components of the fibers, i.e., cellulose, hemicellulose, lignin, pectins, waxes, and fats. However, the spectrophotometric separation of individual compounds from the spectrum is not possible due to the presence of the same functional groups.

According to the literature, both cellulose, hemicellulose, and pectins belong to the group of polysaccharides whose molecules are linked by glycosidic bonds, visible in the 896 cm^−1^ wave number range [59,60]. Our previous research also confirmed the presence of a band at 968 cm^−1^ originating from the 1.4 glycosidic bonds for both flax and hemp fibers, as shown in Figure 7 and Figure 8. In cellulose, the molecules are linked by 1,4-β-glycosidic bonds, whereas in hemicellulose and pectin, they are linked by 1,4-β and 1,3-β-glycosidic bonds.

Lignin is a polymer, monomers of which are organic compounds derived from phenolic alcohols. The most characteristic bands for lignin are symmetric, stretching vibrations for the C=C bond derived from the aromatic ring at 1509 cm^−1^ [59,60,61]. The characteristic bands for lignin also include symmetric deformation vibrations derived from the CH_3_ bond in the 1361–1367 cm^−1^ range. These two bands were also confirmed by our own studies on flax and hemp fibers, as shown in Figure 7 and Figure 8. The studies showed that the occurrence of these bands in the wavelength range of 1361–1367 cm^−1^ for C-CH_3_ symmetric vibrations and 1507 cm^−1^ for the vibrations of C=C stretching of aromatic groups, visible under magnification.

For pectin, the most characteristic band is a stretching vibration derived from the COO ester group bond in the 1428 cm^−1^ range. This region is also observed in the spectrum for all tested flax fibers in Figure 7 and for hemp fibers in Figure 8, regardless of the extraction method used.

In addition to the above bands observed in the spectrum of the fibers tested (flax and hemp), an analysis of the spectra showed common bands in the following wave number regions: 917–1186 cm^−1^ (C-O-C bending and C-O stretching), 1249 cm^−1^ (C-O stretching), 1204 cm^−1^ (CH bending), 1315 cm^−1^ (CH_2_ shear), 1335 cm^−1^ (OH bending), 1464 cm^−1^ and 1473 cm^−1^ (C-CH_3_, C-CH_2_ deformable), 1454 cm^−1^ (OH bending) and 1620–1641 cm^−1^ (OH stretching) for adsorbed water, 1738 cm^−1^ (C=O stretching), 2853–2921 cm^−1^ (CH and CH_2_, stretching) and 3100–3600 cm^−1^ (OH, stretching). The characteristics of the identified transmission bands present in the ATR-FTIR spectra, together with information on the fiber components from which they originate, for all the fibers tested are shown in Table 8.

All the functional groups identified above in the spectra for all the fibers tested have been confirmed by many researchers [59,60,61,62].

ATR-FTIR tests showed that the lowest transmittance signal intensity was found in osmotically degummed flax fibers and, in the case of hemp, fibers obtained via the water retting of straw compared to other fibers, as shown in Figure 7 and Figure 8. This indicates a lower content of chemical components of the fibers on its surface. 

**Figure 9:**
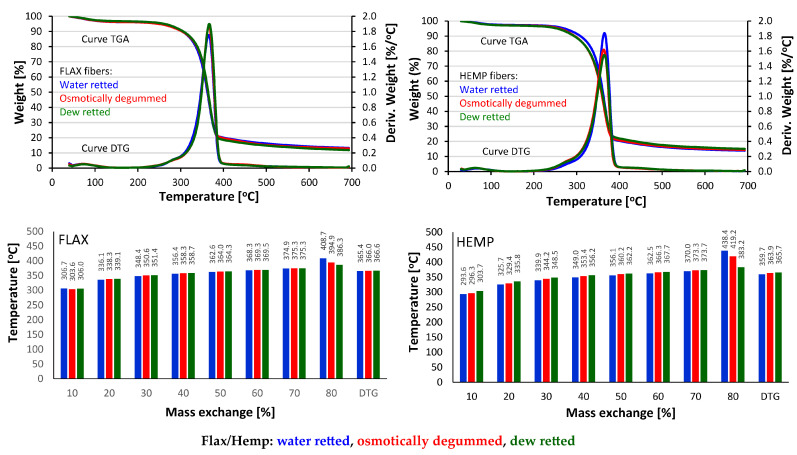
Thermal properties of flax and hemp fibers.

Thermal fiber decomposition tests have shown that this decomposition is in the temperature range of 230 to 530 °C for all the fiber samples tested (Table 9, Figure 9). During the thermal decomposition of all the flax fibers tested, a one-step fiber decomposition was observed, which is the TGA curve.

According to many authors, in the pyrolysis of flax and hemp fibers, four sub-stages of fiber breakdown can be distinguished, as shown by the DTG curve, which is closely related to the chemical composition of the fibers [62,63,64,65,66]. The first decay sub-stage is at about 100 °C and is attributed to the evaporation of water adsorbed into the fibers. The second sub-stage of slight mass loss is in the range of 230–300 °C and is attributed to the degradation of hemicellulose in the fibers. The third decay sub-stage is at a temperature of 300–400 °C and is attributed to the degradation of cellulose in the fibers. As reported by Poletto et al. [65], the crystalline regions of the cellulose molecule improve the thermal stability of lignocellulosic fibers. In contrast, the degradation of cellulose [62] occurs by breaking the glycoside bond in the chain form of glucose. The last, fourth, sub-step corresponds to the degradation of lignin, which occurs in the temperature range from 410 °C to 600 °C [63,65], and, according to Kim et al. [66], the process is slow and starts as early as 250 °C.

Fiber pyrolysis studies have shown that the thermal decomposition characteristics of flax samples obtained using tested degumming methods are very similar. At about 100 °C, there is a few percent loss in the mass associated with water evaporation, and then at a temperature of about 270 °C, the process of thermal degradation of the cellulose fibers material begins, which ends after reaching 390 °C. Differences during individual thermograms and in the amount of solid residue after pyrolysis are negligible. The maximums of the DTG curves are also located in similar temperature ranges.

All hemp samples tested have a similar character—one-stage fiber decomposition with prior water separation. Only osmotically degummed fibers begin the decomposition process about 10 °C earlier than the others. There are also differences in the amount of solid residue after the pyrolysis process, and the osmotically degummed hemp sample is characterized by a higher amount of solid residue up to about 4% compared to the dew-retted fibers. This is most probably related to a much higher content of minerals in the hemp sample that is osmotically degummed (Table 6) since the chemical composition of flax and hemp includes both organic and mineral compounds [1,8,9,67].

Simultaneously, for thermal fibers decomposition studies, the Fourier transform infrared (FTIR) released gases were identified. Spectrum analysis was performed based on the four sub-stages discussed, i.e., water evaporation, hemicellulose, cellulose, and lignin decay. Spectra obtained for furnace temperatures of 100, 300, 366, and 450 °C were analyzed.

**Figure 10:**
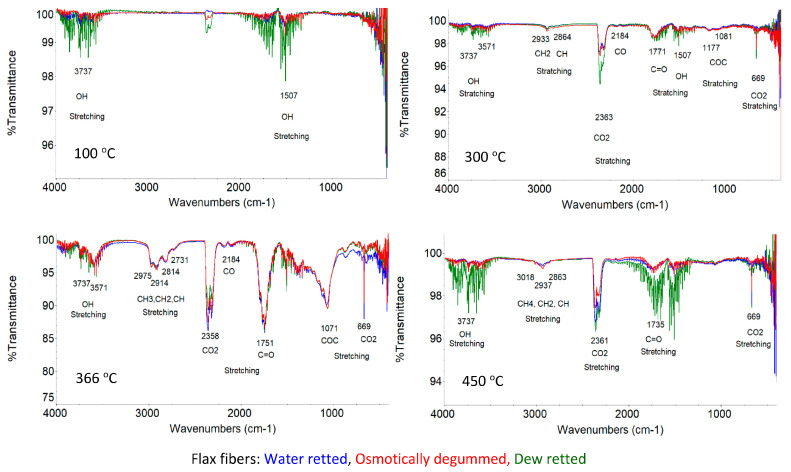
FTIR spectra of released gases during the pyrolysis of flax fibers.

**Figure 11:**
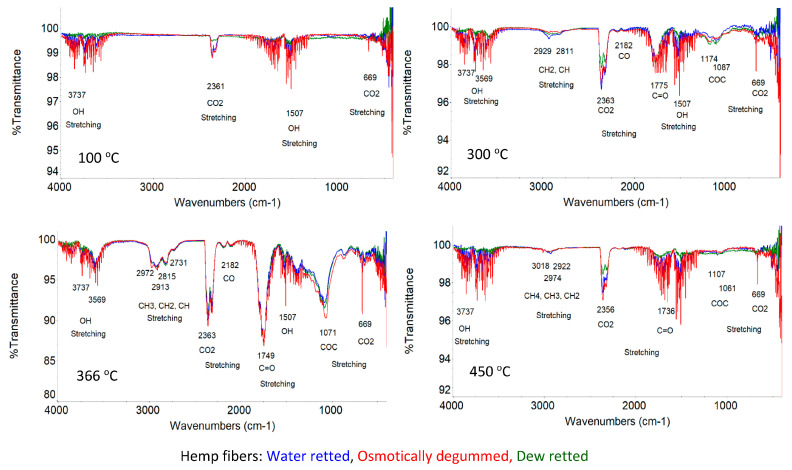
FTIR spectra of released gases during the pyrolysis of hemp fibers.

The detailed analysis of the spectra identified gas release for the different stages of decomposition, for both flax and hemp fibers, are as follows:

I sub-stage—water evaporation, for which the characteristic OH bond stretching bands at 3737 cm^−1^ and 1507 cm^−1^ are visible (Figure 10 100 °C and Figure 11 100 °C).

II and III sub-stages—include hemicellulose (II) and cellulose (III) decomposition, for which the following gases have been identified:

- Water vapor, as in the first stage of water evaporation (Figure 10 300 °C and 366 °C and Figure 11 300 °C and 366 °C);
- Carbon dioxide, for which the characteristic stretching bands for the CO bond at 2363–2356 cm^−1^ and 669 cm^−1^ are visible (Figure 10 300 °C and Figure 11 366 °C);
- Carbon monoxide, for which the characteristic occurrence of a band for CO binding is at 2184–2182 cm^−1^ (Figure 10 366 °C and Figure 11 366 °C);
- Acetic acid and formic acid, for which stretching vibrations of the OH bond are observed at 3571–3569 cm^−1^, stretching vibrations of the carbonyl group for the C=O bond at 1775–1749 cm^−1^, stretching vibrations for C-O acids at 1177–1071 cm^−1^, and stretching vibrations of the -CH_3_ bond at 2974–2972 cm^−1^ (Figure 10 300–366 °C and Figure 11 300–366 °C);
- Formaldehyde, for which vibration bands are observed for the aldehyde group of the CH bond at 2863–2731 cm^−1^, CH_2_ at 2933–2913 cm^−1^ and vibration of the carbonyl group of the C=O bond at 1775–1736 cm^−1^ (Figure 10 366 °C and Figure 11 366 °C).
- IV sub-stage—lignin decomposition for which the following gases have been identified:
- Carbon dioxide, carbon monoxide, water vapor and formaldehyde (Figure 10 450 °C and Figure 11 450 °C);
- Methane, for which CH_4_ bond stretching vibration bands are observed at 3017 cm^−1^ (Figure 10 450 °C and Figure 11 450 °C).

According to the authors [67], the products of thermal decomposition of lignocellulosic components are water vapor, carbon dioxide, carbon monoxide, formic acid, acetic acid, methyl alcohol, formaldehyde and phenol. Our previous work [68] showed no phenol release, whereas it did show methane release.

**Figure 12:**
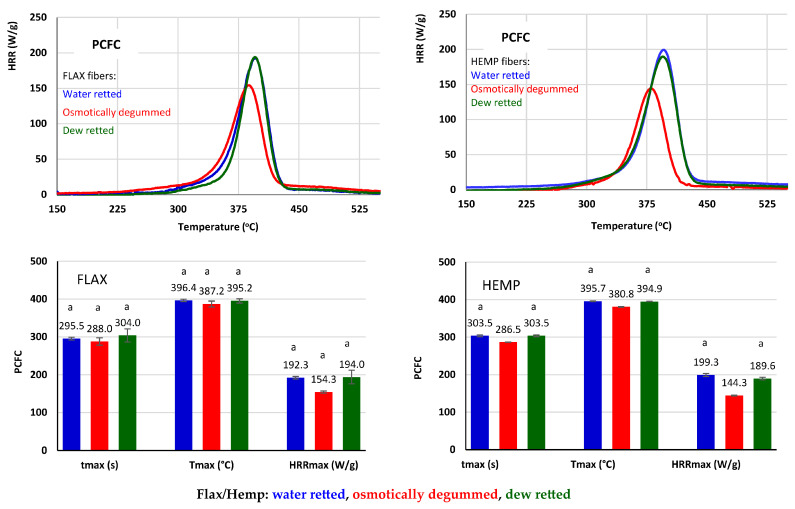
Flammability results of flax and hemp fibers. Results are expressed as mean ± standard deviation (SD), n = 2. Lowercase letters represent the group for which the mean values do not differ statistically at the assumed significance level.

Studies on the combustion intensity of flax and hemp fibers have shown that the rate of fiber combustion varies depending on the method of fiber extraction (Figure 12).

The maximum heat release rate (HRRmax) was reached for dew-retted flax fibers. For the water-retted fibers, a similar result was obtained: HRR decreased by 1.75 W/g, whereas the temp increased by 1 °C in a shorter time of 8.5 s compared to the dew-retted fibers. The lowest HRRmax value was obtained for the osmotically degummed fibers, which was more than 20% lower compared to the dew-retted fibers.

For hemp, the maximum heat release rate (HRRmax) was reached for the water-retted fibers (Figure 12). For the dew-retted fibers, the HRR decreased by 9.7 W/g, whereas the temperature remained the same for 0.8 s less time compared to the water-retted fibers. The lowest HRRmax value was obtained for the osmotically degummed fibers, which was more than 23% lower compared to the water-retted fibers, as statistically confirmed.

The results indicate that the flax and hemp fibers obtained via the osmotic degumming of straw are characterized by reduced flammability and thus a higher flame retardancy compared to fibers obtained using other methods. According to the authors [69], the burning rate is influenced by the adhesion capacity between the fibers and the polymer matrix. It can, therefore, be concluded that the fibers obtained via osmotic degumming straw show a higher adhesion capacity to the polymer matrix compared to other straw degumming methods.

## 4. Conclusions

The results of this study proved that the quantity and quality of the flax (*Linum usitatissimum* L.) and hemp (*Cannabis sativa* L.) fibers change based on the fiber extraction method used. The tests used the osmotic degumming, dew-retting, and water-retting methods.

This paper shows that for both flax and hemp, the highest fiber content in straw can be obtained via osmotic degumming compared to straw in the field and retting in water. The evaluation of fiber quality showed that for both flax and hemp straw, the dew-retting method produced grey-colored fibers, which is the result of the action of fungi. On the other hand, the water-retting and osmotic degumming methods yielded light-colored fibers, which is the result of the action of bacteria, for water retting, and water for osmotic degumming.

The study of the morphological structure of the fibers showed that hemp fibers, as well as flax fibers obtained via osmosis, are characterized by better divisibility of fiber bundles, as confirmed by the cross-sectional view, and less impurities, as confirmed by the longitudinal view, compared to other fibers. The results of the metrological evaluation showed that the osmosis, dew-, and water-retting methods produced similar fiber linear mass values between 0.3 and 0.4 tex. However, for the hemp fibers, the osmosis method yielded the lowest linear mass compared to the dew- and water-retting methods. This results in more divided fibers. Investigations of the tenacity of the fibers tested showed that the fibers obtained using the water-retting method were characterized by the highest tenacity, whereas the fibers obtained using the field straw method had the lowest tenacity.

An analysis of the chemical composition of flax fibers showed that fiber degumming in water, such as water retting and osmotic degumming, resulted in better removal of non-cellulosic components from the fibers than dew retting. For hemp, this effect was not clear.

Flax fibers obtained via dew retting had the highest hygroscopicity of 65% moisture content, whereas those obtained via water retting had the lowest hygroscopicity. On the other hand, hemp fibers obtained via water retting were characterized by the highest hygroscopicity, whereas those of osmotically degummed fibers were the lowest. It has also been shown that for both flax and hemp fibers, the hygroscopicity of the fibers increased with increasing linear mass.

The research showed that the structural analysis (ATR tests) of all the fiber samples tested were similar and typical of flax and hemp. In all tests, flax and hemp fibers were identified with the same bands corresponding to the vibrations of functional group bonds, such as OH, C=O, C=C, COO, CH, CH_2_, CH_3_, COC, and CO. The tests show that the mentioned functional groups originate from the chemical components of the fibers, i.e., cellulose, hemicellulose, lignin, and pectin, as well as waxes and fats, and are closely related to each other.

The measurement of thermal stability showed that the thermal resistance of flax fibers is comparable to that of falls in the temperature range from 230 to 530 °C, regardless of the fiber-obtaining method used. In the case of hemp fibers, only the osmotically degummed fibers began the decomposition process about 10 °C earlier than the fibers extracted via the dew- and water-retting methods. Gases such as carbon dioxide, carbon monoxide, water vapor, acetic and formic acid, formaldehyde, and methane were identified in all the fibers tested during thermal decomposition, regardless of the fiber extraction method used. Flammability tests have shown that for both flax and hemp, fibers obtained using the osmotic degumming method have the lowest HRRmax value compared to fibers obtained using the dew-retting and degumming methods. This results in a reduction in the flammability of the fibers and, consequently, an increase in the fire resistance of the fibers.

## Figures and Tables

**Table 1:** Methods of obtaining fibers from bast fibers plants [1,2,3,4,5].

Mechanical Processing	Biological Methods			Chemical Degumming	Physical Methods
	Water Retting		Dew Retting		
	Natural Water Reservoirs	In a Basin			
Decortication, Scutching, Hackling, Carding, Cottonisation.	River, Ponds, Pits.	Cold water Warm water With/without aeration Fluid recirculation Double retting Enzymatic With additive Nitrogen nourishment Ferments Bedding made of retted fibers	Using: Desiccants Breaking straw in the root section Nitrogen media	Acetylation Acids: oxalic, sulphuric Alkali: sodium hydroxide) Combination of acids and alkali Oxidizing agents: hydrogen peroxide)	Steam explosion High-frequency electromagnetic radiation Electro osmosis Ultrasound Osmosis

**Table 2:** Process conditions of degumming flax and hemp fibers from straw.

Process Conditions	Flax	Hemp
Temperature, °C	30	30
Time, h	72	144
Ultrasound, kHz	30	30
Flow speed, dm^3^/min	30	30
Batch mass, kg	15	20

**Table 3:** Results of the qualitative assessment of flax and hemp straw.

Straw	Length		Thickness	Color			Health	Attitude	Straw Class
	Technical	Total		Yellow	Green-Yellow	Green			
	cm	cm	mm	%	%	%	%	%	
FLAX	67	74	1.23	100	-	-	100	100	I
HEMP	184	199	6.4	20	60	20	100	100	I

**Table 4:** Mass of flax and hemp fibers.

Fibers	Mass of Extracted Fibers					
	Total		Long		Short	
	%	SD	%	SD	%	SD
FLAX						
Water retted	35.08 ^a^	0.82	25.74 ^a,b^	1.83	9.34 ^a,b^	0.91
Osmotically degummed	38.12 ^b^	0.84	29.64 ^b^	0.91	8.48 ^a^	0.83
Dew retted	36.25 ^ab^	1.28	24.58 ^a^	1.92	11.66 ^b^	1.16
HEMP						
Water retted	28.89 ^a^	0.96	17.78 ^a^	0.96	11.11	0.96
Osmotically degummed	34.44	0.96	28.33	1.67	6.11	0.96
Dew retted	26.67 ^a^	1.67	18.33 ^a^	1.67	8.33	0.00

Results are expressed as mean ± standard deviation (SD), n = 3. Lowercase letters represent the group for which the mean values do not differ statistically.

**Table 5:** Metrological evaluation of flax and hemp fibers.

Fibers	FLAX				HEMP			
	Tenacity		Linear Mass		Tenacity		Linear Mass	
	cN/tex	SD	tex	SD	cN/tex	SD	tex	SD
Water retted	24.15 ^a^	2.59	0.3 ^a^	0.08	7.52 ^a^	1.37	1.1 ^b^	0.05
Osmotically degummed	20.84 ^a^	1.82	0.3 ^a^	0.07	7.51 ^a^	1.54	0.8 ^a^	0.18
Dew retted	13.88	1.45	0.4 ^a^	0.07	4.59	0.74	1.0 ^a,b^	0.15

Results are expressed as mean ± standard deviation (SD), n = 5. Lowercase letters represent the group for which the mean values do not differ statistically.

**Table 6:** Chemical evaluation of flax and hemp fibers.

Fibers	Content of:									
	Waxes and Fats		Lignin		Pectin		Cellulose		Hemicellulose	
	%	SD	%	SD	%	SD	%	SD	%	SD
FLAX										
Water retted	1.42 ^a^	0.05	3.07 ^a^	0.11	1.16 ^a^	0.15	74.83 ^a^	0.68	14.43 ^a^	1.22
Osmotically degummed	1.84	0.06	3.49 ^a^	0.20	1.44 ^a^	0.16	74.87 ^a^	0.68	14.57 ^a^	0.28
Dew retted	1.50 ^a^	0.09	5.29	0.60	2.50	0.20	72.29 ^a^	0.93	18.93	0.42
HEMP										
Water retted	0.19 ^a^	0.00	2.78	0.12	0.66	0.06	70.36 ^a^	0.62	21.22	0.12
Osmotically degummed	0.21 ^a^	0.01	4.25 ^a^	0.03	1.07	0.17	70.13 ^a^	0.45	15.99	0.13
Dew retted	0.26	0.01	4.11 ^a^	0.01	2.27	0.29	71.42 ^a^	0.66	18.96	0.13

Results are expressed as mean ± standard deviation (SD), n = 3. Lowercase letters represent the group for which the mean values do not differ statistically.

**Table 7:** Hygroscopicity of flax and hemp fibers for 65% and 100% relative air humidity.

Fibers	FLAX				HEMP			
	65%		100%		65%		100%	
	%	SD	%	SD	%	SD	%	SD
Water retted	8.80 ^a^	0.04	11.48 ^a^	0.24	10.85 ^a^	0.21	13.96 ^a^	0.52
Osmotically degummed	8.94 ^a^	0.00	11.78 ^a^	0.07	9.81	0.12	13.14 ^a^	0.33
Dew retted	9.44	0.05	11.94 ^a^	0.18	10.62 ^a^	0.03	13.32 ^a^	0.19

Results are expressed as mean ± standard deviation (SD), n = 2. Lowercase letters represent the group for which the mean values do not differ statistically.

**Table 8:** The characteristics of the main absorbance spectra in FTIR of flax fibers.

Bond	Vibration Type	Wavenumber [cm^−1^]	Remarks
O-H	Stretching	3100–3600	Cellulose, hemicellulose, lignin, pectin
C-H, C-H_2_	Stretching	2853–2921	Cellulose, hemicellulose, lignin, pectins, waxes, and fats
C=O	Stretching	1730–1738	Carboxylic acids, aldehydes, esters (pectin, lignin, waxes and fats)
O-H	Stretching	1620–1641	Adsorbed water
C=C	Stretching	1509	Pick characteristic for lignin
O-H C-H_3_ and C-H_2_	Bending Deforming	1454 1464–1473	Adsorbed water Lignin and cellulose, hemicellulose, pectins, waxes, and fats
COO	Stretching	1428	Acids (pectins)
O-H	Bending	1335	Cellulose, hemicellulose, lignin, pectin
CH_2_	Scissoring (bending)	1315	Cellulose, hemicellulose
C-O	Stretching	1249	Hemicellulose, pectins
C-H	Bending	1204	Flax, hemp
C-O-C C-O	Bending Stretching	917–1186	Cellulose, hemicellulose, pectin
1,4-β-Glycosidic bond	Stretching	896	Cellulose, hemicellulose, pectin

**Table 9:** Thermal properties of flax and hemp fibers.

Fibers	Decomposition of the Main Fibers Component						Residual Mass at T_700_ °C	
	T_Int._		T_Fin._		Mass loss			
	°C	SD	°C	SD	%	SD	%	SD
FLAX								
Water retted	296	2.04	385 ^a^	1.63	82.64 ^a^	1.63	13.93	0.24
Osmotically degummed	300 ^a^	2.23	388 ^b^	1.41	81.41 ^a^	0.90	13.05	0.23
Dew retted	300 ^a^	2.41	386 ^ab^	2.09	82.01 ^a^	0.84	11.96	0.41
HEMP								
Water retted	300 ^a^	1.73	393 ^a^	1.86	83.55 ^a^	1.47	12.53	0.13
Osmotically degummed	290	1.95	385	2.01	80.09 ^a^	2.24	15.35	0.12
Dew retted	300 ^a^	2.15	390 ^a^	2.33	83.64 ^a^	2.98	11.93	0.12

Results are expressed as mean ± standard deviation (SD), n = 5. Lowercase letters represent the group for which the mean values do not differ statistically at the assumed significance level.

## Data Availability

Data are contained within the article.
